# Space, time and memory: tales from a long road

**DOI:** 10.1007/s10339-018-0879-0

**Published:** 2018-08-21

**Authors:** Alan Dix

**Affiliations:** Computational Foundry, Swansea University, Swansea, UK

## Abstract

Points, lines and surfaces are the basic elements of Euclidean geometry. In this paper, accompanying a keynote at ICSC 2018, we will explore how, in physics, cognition and our lived experience, it is often better to think in terms of interconnected threads than an evolving state of the world now. In physics ‘now’ is an illusion, merely a convenient construction, each particle and person is more like an independent strand in space–time, and similarly, in our minds, strands of memories from different roles and contexts flow almost independently. Paths create lines on the map and may be inscribed as signs in the landscape, but our journeys along paths create temporal threads that interweave as we meet along the way. This paper draws on my research over many years on time and user interaction and also my personal experiences during a thousand-mile walk around the periphery of Wales in 2013.

## Introduction

All roads lead to Rome: the Milliarium Aureum in the Forum marked the point from which distances were measured along the long roads that led in all directions. However, this is not simply a statement about one end point; it says *to*, not *from*. Roads are spatial, but journeys are temporal, with a beginning and an end. Furthermore, in Imperial times and for a millennium after, Rome was the centre of power, temporal and spiritual; the ‘to’ makes clear that one’s eyes should be cast towards its heart. Our conceptions of space and time are intimately interwoven with the social fabric of human life.

In 1996, at the AVI’96 conference in Gubbio, I gave my first keynote, which considered how sight, sound and smell create different cuts through space and time and call on memory in different ways to fill the void in current sensation. Twenty years on, at Talis, I studied the changing nature of physical text in a digital world. The written word takes a snaking line across the space of the page and through the volume of a book, preserving, prompting and promulgating memory.

During the Dark Ages, the laborious writing of Celtic monks on the Atlantic fringe was crucial to the continuity of knowledge. In 2013 I took my own winding route round the edges of Wales, exploring viscerally the relation between space, time and memory. This paper will link lessons from that one-thousand-mile path and 30-year study of time in user interaction.

## In the eye of the beholder

Time is notoriously difficult to define.

Augustine of Hippo (AD 401) famously asked, ‘*What then is time?*’, and answered himself, ‘*If no one asks me, I know: if I wish to explain it to one that asketh, I know not’*.

It is obvious and yet an enigma.

Even today physicists struggle with an apparent contradiction. On the one hand, there is a clear large-scale ‘arrow of time’, the relentless increase in entropy. Yet this is set against the timeless quality of both quantum mechanical and relativistic accounts, within each of which all processes are reversible, and the distinction between past and future is no more than between left and right.

Augustine goes on to ask, ‘*But time present how do we measure, seeing it hath no space?’*

Of course, post-Einstein we see space and time as more intimately connected, capable of twisting into one another as easily as a compass needle shifts from south to west. Yet cognitively, in our lived human experience, seconds and metres are still as incommensurate as they were to Augustine 1600 years ago.

In any statement, the assumptions that underlie it are often more fascinating than the subject itself. Augustine’s ‘*how do we measure it?’* casts the problem as being the space-less nature of time, but thereby assumes that if time had spatial dimensions it would be unproblematic.

Is it really so clear?

Modern physics suggests that time is perhaps no more complex than space, but equally that space is no less complex than time.

Andrew Parker (2004) argues that the Cambrian explosion was due to the evolution of the eye. Before the eye, there were ways to sense at a distance; indeed a mole, while blind, can still navigate through the sense of smell. However, the ability to see at distance revolutionised both predator and prey.

At AVI’96 I explored the ways in which different senses give different ‘cuts’ through time and space (Dix 1996): vision cuts through space, telling us about what is happening now as far as the eye can see; for a dog, smell cuts through time, telling it about everything that has happened here back through the past until the scent fades; and for an echolocating creature like a bat, there is something in between, where closer things are known instantly, more distant ones after a delay.

Today, half of the human brain is dedicated to visual processing, and this primacy of vision can effectively blind us to the world view it creates. Indeed, the previous sentence includes two metaphorical uses of ‘vision language’ in day-to-day speech. Crucially our sight gives us a sense that space is simple, taken in at a glance. However, consider a sightless worm: the lawn that we survey in an instant is experienced predominantly temporally, traversed over hours. Indeed, in the days before satellites, distances too far to see or hidden by forest or hill would be measured not in metres or miles, but in the number of days’ walking it took to reach them.

There is an argument that both physically and cognitively time is more fundamental than space.

## Not now

One of the fallacies of vision is the illusion of now.

The near-instantaneous travel of light means that we perceive a single glance as encompassing the world at a moment. This perception of ‘now’ in the immediate environment, the ‘here and now’, is perhaps as old as vision itself, but precise measurement of ‘now’ at a distance has emerged far more recently, and it is not clear when the idea of ‘now’ as a universal global snapshot emerged.

From early times sundials and sun sticks were used to measure the passing of time through the day. Later, town clocks replaced the sundial as the primary means to measure time, but were synchronised to the same movements of the sun: twelve noon was when the sun was at its zenith.

One of the revelations of a spherical earth, whether in a geocentric or heliocentric universe, is that the local sun time is not universal: noonday in Rome is more than an hour before noonday in Swansea. North–south this sphericity allowed sailors to measure their latitude through the azimuth of the noonday sun, but for many years longitude, east–west location, proved elusive.

The importance of this was sufficient that from the mid-sixteenth century national prizes were offered for the person who cracked the problem of longitude. However, it was not until 1740 that John Harrison’s highly accurate chronometer solved the problem (Sobel 2005). The chronometer was set to a fixed time, notably the time at Greenwich, home of the British fleet, and kept time accurately over the length of a voyage. The further west you go, the later the sun rises, and the later it reaches its zenith. Thus, by simply noting the chronometer time at local noonday, it was possible to work out how far east or west you were; each hour delay in noontime corresponds to 15 degrees of longitude.

The Harrison chronometer not only meant that space was measured using time, but it also provided a highly accurate way of saying ‘now’ globally.

In the nineteenth century, railway companies began to find the local time of the town hall clock inconvenient. If trains were to adhere to strict timetables, it became important that train guards were not constantly adjusting their watches to fit with local times. Initially each company created their own unified time, and eventually national time zones were established, overriding local celestial time. In factories clocks were also installed, not just for punctuality of timetables, but to ensure a regulated workforce: the patterns of summer and winter, dusk and dawn, ignored in the race for efficiency and control.

Time is not simply a matter of physics but an instrument of social and political engineering. Indeed, in 1940 General Franco shifted Spain’s clocks to Berlin time even though the sun rises more than an hour later in Madrid.

However, ‘now’ is an illusion, or at best an invention.

Newtonian physics is rather like lasagne. Imagine each layer is the state of all things in space at a single moment in time; below it lie past sheets of now, and above it fresh sheets are added each moment. The laws of physics specify how each layer gives rise to the next.

Einstein’s genius was in realising that there is no ‘now’, no universally meaningful sheet of present. Instead, relativity is more like a tangle of spaghetti: each of us carries our own clock, our own time, and our knowledge of space is limited to the proximate; we are but a thread through time–space. Sometimes our spaghetti threads lie alongside one another, and then we share our here and now, our clocks progress in synchrony, but then, when our threads diverge there is no reason for our clocks to keep similar time.

Although we each carry our own time, we can attempt to create a universal now. Einstein envisaged doing this through communication: send a light beam from Swansea to Rome and then, when it arrives, send one straight back again. If Swansea records the light beam leaving at 12 noon precisely and receives the return beam at 12:00.014, we can assume that the light beam reached Rome precisely half-way between, at 12:00.007. By doing this, every point in space and time can be given a precise Swansea time, and each moment of Swansea time creates a hypothetical ‘now’.

This is effectively how time can be calibrated for practical purposes and, at day-to-day accuracies, our definitions of ‘now’ using this method agree. However, this is only an approximation. If our relative velocities differ, the definitions we construct of ‘now’ also differ, albeit only noticeable when we travel at speeds that are substantial fractions of the speed of light. Your lasagne sheets of hypothetical ‘now’s’ criss-cross my sheets. Furthermore, each time you accelerate or slow down, your sheets of hypothetical now, which spread out across the universe, swing back and forth like a child playing planes, running with arms outstretched. As we bank and turn, the hypothetical times we have for distant places may shift back and forth by aeons.

Of course for most speeds and scales, it is possible to construct universally accepted ideas of ‘now’, but the physical lack of ‘now’ does call into question the extent to which this is a universal human concept. Psychology has been based predominantly on so-called WEIRD subjects (western, educated, industrial, rich, democratic), but comparative studies have found that what might be thought to be universal cognitive and perceptual phenomena are, in fact, sometimes shaped by culture (Henrich et al. 2010).

Is this true of ‘now’? Would a Neolithic hunter-gatherer have been able to think during the day, ‘I wonder what is happening back at the cave *now*.’?

## Paths and journeys

In 2013 I walked the thousand-mile journey around the periphery of Wales: the mountains and meandering rivers of the eastern borderlands, and the fractal folded coastline of the north, west and south (Dix 2013a, b). The journey skirted the edges of what were once the world’s largest ports (the sea is the ultimate highway), past derelict industrial sites and of course rural communities at the economic and digital edges.

The *route* followed two existing *paths*: the long-standing Offa’s Dyke long-distance path, which runs up the eighth-century border between Wales and England, and the then new Wales Coast Path, the first complete national coast path in the world.

The paths are drawn as lines on maps, but also *inscribed in the landscape* itself: arrows, finger signposts and small roundels on gateways and stiles. Crucially, on the more heavily trodden parts of the paths, the way to go is implicit in worn *tracks* across the ground.

Tim Ingold (2007) highlights the modern Cartesian obsession with points in space: cities, towns, landmarks. The lines, the paths and routes between are seen as merely means of traversal. ‘*All roads lead to Rome’*—it is Rome, the destination, that is prime, not the road on which your feet tread.

Some of the wooden finger posts along the Offa’s Dyke Path and Wales Coast Path do give distances to the next major place along the way. Of course, on walking paths ‘major’ may mean a tiny village, hilltop, bay or headland, but these still appear at first to share the Cartesian-centric focus of the motorway sign.

There is indeed an element of this: when you are looking for the next place to find food, or where you plan to sleep that night, these destinations are important. However, unlike the typical motorway driver you are not travelling the path in order to get to a destination; the destinations are merely the means by which you navigate the path. If you wanted to get from A to B, you would drive or take a bus; the purpose of a long-distance walking path is the walk.

Ingold has studied nomadic tribes for whom, like the leisure walker, the focus on the point, the place, is incongruous and almost irrelevant. Ingold instead suggests foregrounding the line, the connection, the dusty path.

With such a view, Rome might be seen as merely the nexus at which roads happen to meet. This at first seems incongruous: the reason for the Roman roads is precisely to send legions back and forth from the Imperial centre to subdue the periphery and bring back the spoils. They truly are Cartesian lines of connection, cutting across the landscape, drawn arbitrarily across a map, with scant regard for landscape and topography. However, at another level the location of most established cities is precisely because they are at the intersection of routes: river mouths such as the Tiber for water transport, fords across rivers, passes between mountains.

Whether worn by use, drawn on a map or constructed by bulldozer, paths and roads are static and impersonal. Journeys, from the Odyssey, Argonautica and Viking Sagas to the modern travelogue, are often about people, and indeed their personal stories and the land and sea paths that they traverse are as much about significant events as significant places.

On the paths around Wales, signposts were often simply two tiny arrows in opposite directions, saying ‘Offa’s Dyke’ or ‘Wales Coast Path’—one path, two directions, the only way you knew which one to follow was that the other pointed backward along the way you had come. A *path* is static and spatial; a *journey* is dynamic and temporal.

I recall at one point (note the spatial language for a temporal event), before I set off walking but after plans were laid, someone mentioned a place in Pembrokeshire. As the name was spoken I noticed myself instantly thinking of it as ‘late June’—I had already internalised the route and approximate timeline, and for me the spatial organisation of the coast had become a temporal organisation of my own journey along it.

## People along the way

If a journey is personal, then for each traveller, the journey, even when taking the same route, is unique.

Geographers often distinguish space and place, a distinction that has also been adopted within human–computer interaction (Harrison and Dourish 1996). The former, space, is used to refer to the Cartesian location or extent, the point on the map; however, when that location becomes flooded with social and historical significance, it becomes a place. Auge (1995) talks of non-places: shopping malls, service stations and airports, the soulless loci of modernity. Yet these apparently mere points of passage are for some—the shopkeeper, ticket seller or cleaner—places of abode where life is lived. Even for the traveller they may be landmarks, meeting places, or, like the railway platform in *Brief Encounter*, where lovers tryst (Dix and Gill 2019).

Some years ago, I needed to set a design challenge and chose ‘absent presence’ (Dix et al. 2004). There are places, such as art galleries or a town centre at night, where you may be alone, and yet there have been others before you in the same spot, and others will come after you. How do you help people appreciate this absent presence?

**Fig. 1:**
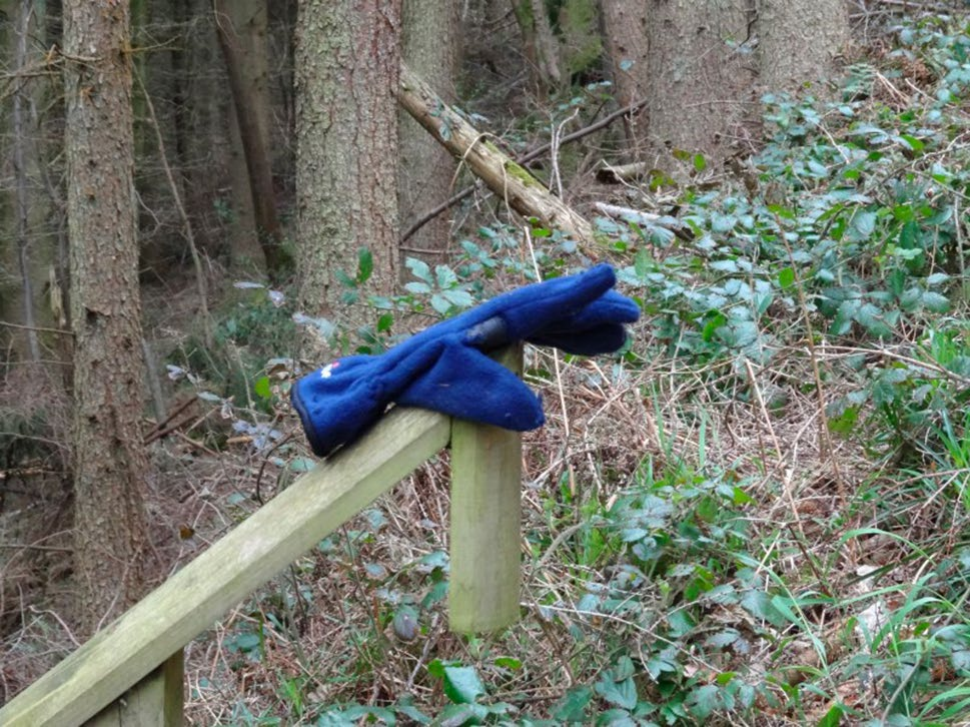
Signs left behind (photograph: author, see Dix 2013a, day 4)

For paths no less than places, people have trod before you; indeed, there are often the signs left behind: a footmark in the mud or a lost glove (Fig. 1). Yet you walk alone—the stories of these other travellers, the journeys that have briefly inhabited the path are at best guessed if ever even envisaged.

Your own traces on the ground fade, the footmarks washed away or overlaid with others, the only remains the slight deepening of a rutted path or the gloss on well-trodden stones. Often the trace that persists is on paper in diaries and journals, or digitally on the web: lines of text and photographs.

On my journey I took 19,000 photographs, approximately one every 100 metres, and wrote more than 150,000 words of blog, approximately one character for every second step. In addition, the walk produced the largest ECG trace in the public domain as well as other data, all time-stamped: personal record and automatic sensing linked through time.

A colleague, Stavros Asimakopoulos, analysed my blogs using grounded theory methods, inductively building concepts and themes from the raw words (Asimakopoulos and Dix 2017). When he first showed me the results, I did not believe him. He said that the major constructs were around social engagement, and yet I knew that for virtually the entire time I had walked alone, some days barely meeting a single person. However, he was right. Although social interactions were only a small part of the temporal experience, in my written reflective account, these human contacts took centre stage.

**Fig. 2:**
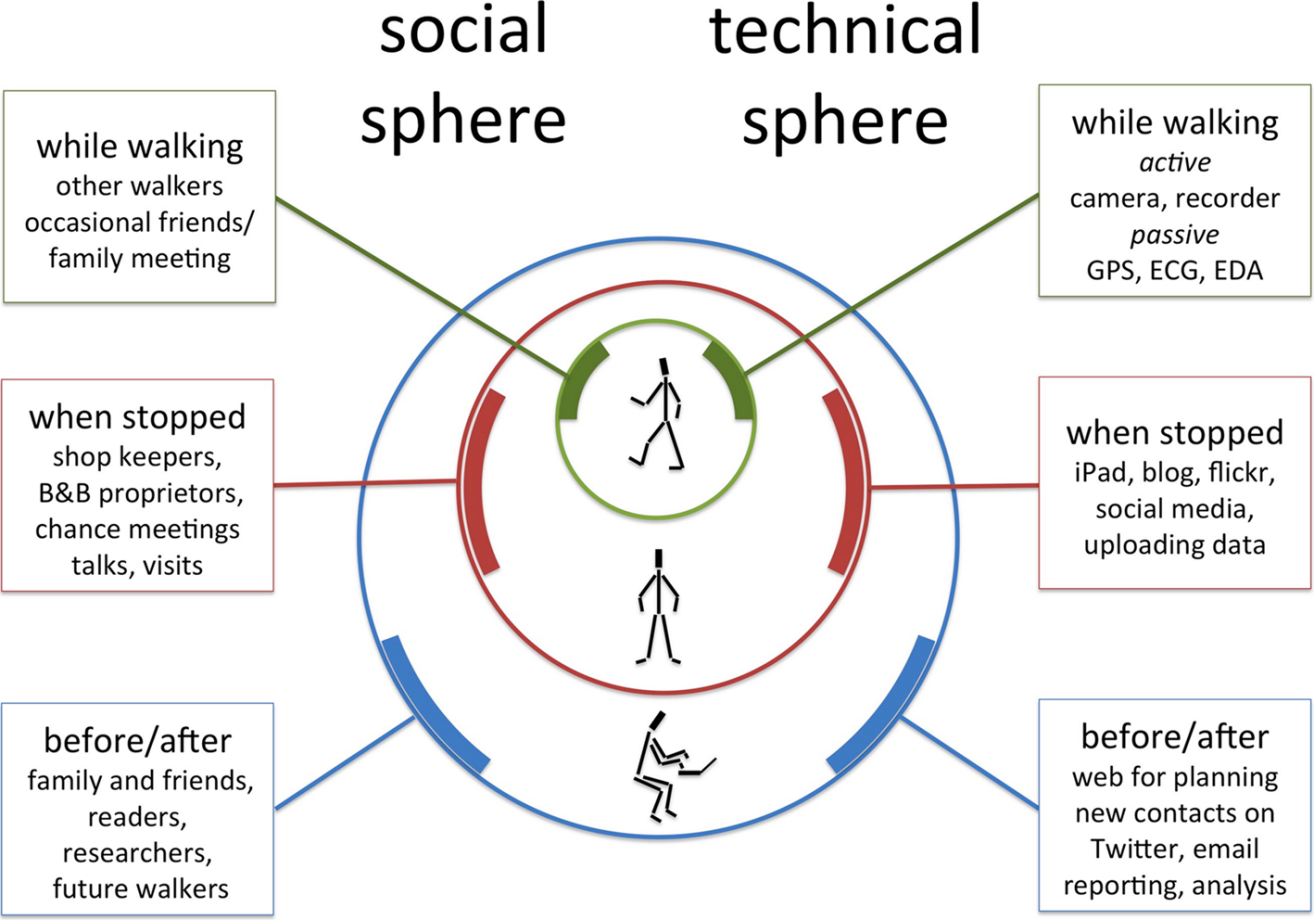
Onion layers of social connections and technology use

On reflection it became clear that while at any moment there was little or no social or technical component to the experience, simply the walking, once I expanded my viewpoint there were onion layers of experience extending into time and space (Fig. 2).

Three broad classes of people became apparent.

*Egocentric* (people of life)—these were my personal contacts (family, friends and professional colleagues), who during the walk were principally connected virtually and remotely.

*Geocentric* (people of the land)—these were the shopkeepers, bed and breakfast owners, or simply local people met in the various villages, towns and cities through which I passed.

*Tribocentric* (people of the way)—these were the fellow walkers and travellers, who I met briefly, a few walking long distances like myself, other simply on days’ out or holidays.

These differ in the ways in which we meet and connect along the way, but the ‘people of the way’ are most interesting in the context of this paper: those with whom we momentarily cross paths, or maybe walk alongside for a while—just like the strands of spaghetti-like existence in relativity.

## Cognition: is all knowledge spatial?

In previous work I have explored the relationship between the ways we build childhood understanding of significant places and the routes between them, and the way in which, as adults, we create interconnected maps of ideas (Dix 2009).

While our visual world is Euclidian (in 2 or 3 dimensions) the world beyond our sightline is one of paths and landmarks, a network, not a map. Space syntax uses these broken sightlines as a way to structure space and finds that distance metrics based on changing sightlines offer a closer match to human activity than those based on Euclidean distance (Hillier 1996). Our larger spatial maps are networks.

Spatial and temporal metaphors are common in day-to-day language (Lakoff and Johnsen 2003) and are also found repeatedly in the language of information: items *in* a data store, building *upon* past knowledge, *navigating* to *locations* on the web. Spatial understanding is clearly far more primitive than abstract concepts; indeed maze running experiments are performed on many kinds of animal, which we would not normally associate with high-level abstract thought. So it is unsurprising that spatial metaphors are powerful. It is less clear whether these are simply parallels, or whether they reflect a deeper neurological reuse of aspects of the brain originally developed for spatial reasoning. However, it does appear that spatial-processing parts of the brain are recruited for mathematical reasoning (Dehaene et al. 1999), so this spatial reuse may be more widespread for other areas of intellectual life.

Whether or not there are neurological underpinnings, at very least the structural parallels between spatial and intellectual *landscapes* offer heuristics to understand broader cognition (Dix 2009).

One example of this is the way linguistic, tribal, administrative and nation-state boundaries often lie along physical barriers such as rivers, sea coasts or mountain ranges. This is not unsurprising, since these form natural defences and natural barriers to movements of people. However, there are often equally substantial topographical features that lie wholly within a single geographic entity. The physical features create obvious places where human boundaries may form, but which particular physical features become borders is tied to specific historical events. Nation boundaries sometimes even lie across open ground, such as the plains of central Europe, albeit that these unnatural, exposed borders are frequently the site of conflict.

This tendency for geopolitical boundaries to reflect natural features, while not being entirely determined by them, has parallels in our intellectual life. The nature of the world imposes or suggests particular conceptual breakdowns, such as by similarity or proximity, but which particular set of features emerges as the high-level ontology for a particular individual, culture or language depends on the exigencies of history. This is evident in the Dyirbal language’s category of ‘*Women, Fire and Dangerous Things*’, which Lakoff (1987) highlights in his book of the name.

## Memory: interwoven threads

**Fig. 3:**
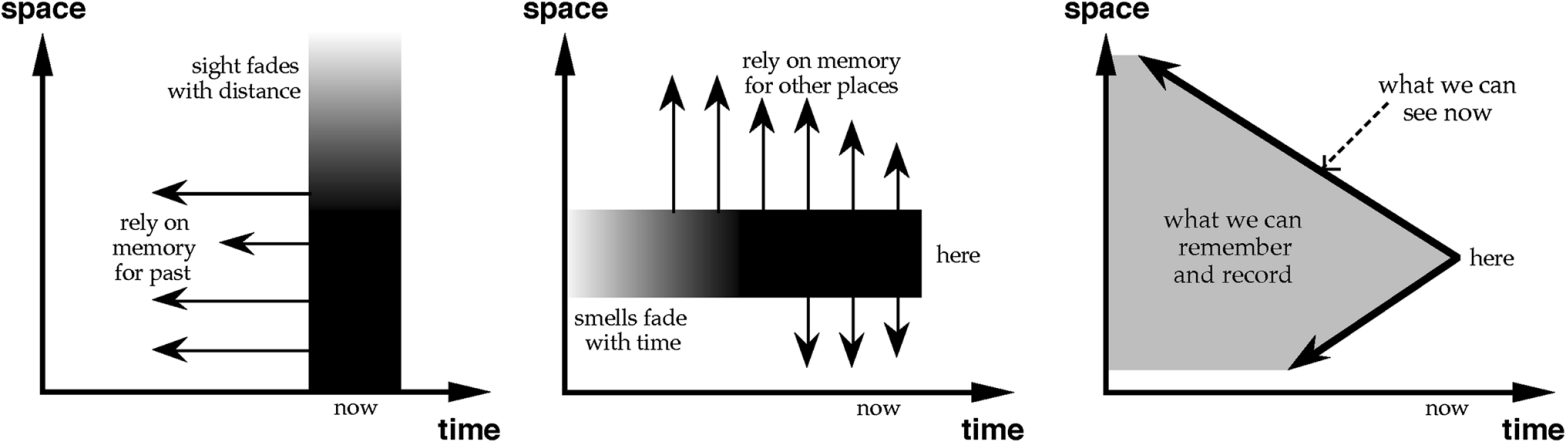
Different senses give different cuts through time, space and memory. Left: Visual perception, centre: Nasal perception, right: Whales and astronomers. (from Dix 1996)

Returning to the sensory cuts through time and space in the AVI’96 keynote (Dix 1996), memory also plays a central role. When a dog smells one tree trunk and then moves to another, its knowledge of the second tree trunk through time is generated directly by its current smell, but its knowledge of the first tree trunk and its history of smell has to be committed to memory. Similarly when we look out we get a (near) instantaneous impression of a wide area of space at an instant, but we rely on our memory for what was happening within our vista at a previous time. That is, a creature of vision determines spatial information with senses and projects backwards in time with memory, but a creature of scent determines temporal information with senses and projects outwards in space with memory (Fig. 3).

In truth all of us are mixtures of the two: as we turn our head we rely on memory to tell us what is behind, and even what appears to be a momentary glimpse of the world ahead is in fact the accumulation of numerous saccades, giving detailed information about different parts of the image, which we unconsciously piece together to give the perception of a visual field. Furthermore, what we seem to see is often a combination of what we actually sense and assumptions built upon previous experience, so that memory constructs the present as much as it reconstructs the past.

In computing we also deal with memory: the state of the machine: registers, stacks and program counters; RAM, hard disks and ‘the cloud’. State is critical, it is our only route to the past, but in coding it is often implicit in variables, the current line of code and call history. Because of this novice programmers often have a less clear model of the state they are creating than physicists brought up on lasagne models of the world. This is crucially important in event-based programming, whether it is user-interface code in a web page, web transactions or low-level concurrent or distributed systems. It is interesting to see the way patterns from state-less functional programming (which therefore needs to deal with state explicitly) are being used in both massive-scale big-data parallelism (MapReduce) and event-driven JavaScript UI code (monads).

There are many parallels between computational memory and human memory. This is partly because they are effectively trying to solve similar ‘problems’ and partly because computer models of memory are often inspired by (assumed) models of human memory. Indeed, in my own previous work with various colleagues we have extended this to include parallels with memory social and organisational systems (Dix et al. 1998, Dix 2002), asking questions such as ‘what happens to the organisational program counter when the lights go out at night?’ These parallels are powerful, but may occasionally be false friends; the largely sequential and linearly structured nature of classical computation may not reflect the more associative nature of subconscious cognition.

In a computer we often model temporal data as time series, with a value recorded at regular points of time, or as events ordered by time of occurrence. Is this the way thought works?

**Fig. 4:**
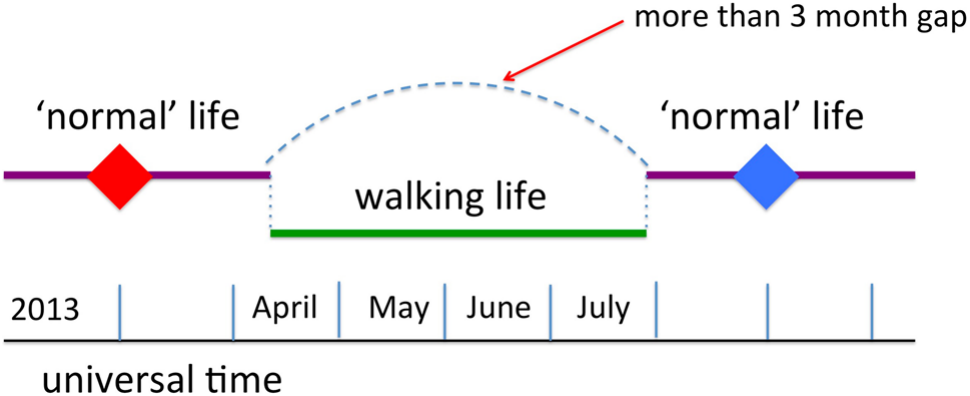
Walking and ‘normal’ life in clock time

I was walking for 3 months (Fig. 4) and after a period I became ‘the walking man’, it was hard to imagine that there had been any existence other than walking or that walking would not go on for ever. However, when the walk finished it was frightening how quickly I got back ‘to normal’—none of the life changing transformations we are led to expect of the grand expedition.

However, for some time afterwards I noticed an odd dislocation of time. One manifestation of this was the way that memories of walking almost seemed to be of a different person. In some ways this was like becoming ‘the walking man’, a shift of personal identity, of habits and expectations. Models of memory often deal with schemas, and the period of walking was clearly a very different schema to day-to-day life, hence a feeling of otherness.

However, the more disturbing dislocation was a broken perception of duration. Time and again I would think that some event had happened recently, in the past few months, only to realise it was half a year ago. There is the classic trope of ‘years flying by’ as you grow older, but this was more dramatic.

**Fig. 5:**
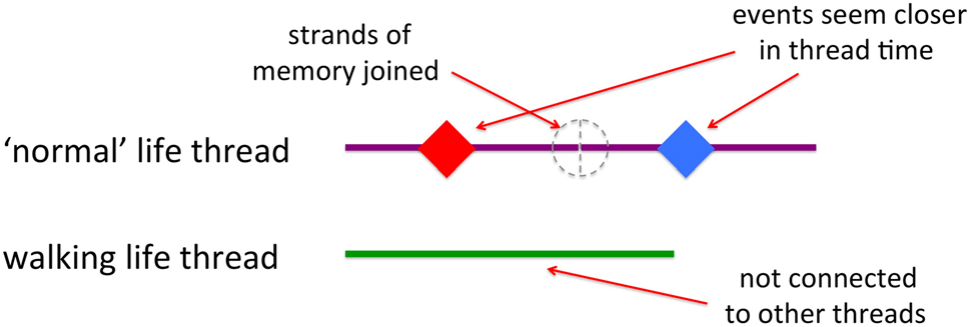
Separate threads of memory

After a while the reason became apparent. I had been living ‘normal’ life and then did something unusual. When I returned to normality my mind had stitched together the time before the walk with the time after, so that I had effectively ‘lost’ 3 months of normal life (Fig. 5). My perception of past time was not based on how far back it was in a universal timescale (even a personal one), but on how far back in ‘normal life’ time. It is rather like visiting friends you haven’t seen for a while, or a much frequented holiday destination; it is as if ‘you have never been away’.

Haliyana Khalid studied photologging sites (like Flickr), where users post, share and comment on photographs. Through this study we became aware of a wider phenomenon, which we called extended episodic experience (Khalid and Dix 2010).

Often, accounts of user experience focus on a specific moment or episode: attending a concert, drinking a bottle of wine, using a word processor. This may include preparation and anticipation before the event, or recollection and reflection afterwards (McCarthy and Wright 2004), but is still focused on the singular event.

The users’ accounts of their photologging suggested a more temporally extended view, where past experiences of photologging, the user’s own reactions to another person’s post or other people’s comments on theirs influenced future decisions; and where the current experience of posting was based on anticipation of future events. These episodes of experience thread together, like pearls on a string.

In Einstein’s physics we saw that we are each like a single strand of spaghetti, a thread of existence.

Cognitively it seems our memories are more like multiple strands, a thread for each different kind of existence: one for home life, one for work, one for holidays, one for our journey to work, etc. These are not aligned by universal time; they are not like the GPS, ECG, accelerometers and EDA traces of my walk data, connected by timestamps. Instead they are almost independent, each carrying their own duration, their own past and future, connected only through points where two threads share a common event.

Benford et al. (2009) talk about *trajectories*, the way we may each experience the timeline of a game or dramatic narrative at different paces so that when you or I read the same book, we experience the same time point in the story at a different personal time, or even in a different order.

It seems that our own internal lives are not so different. The threads of our memory are bound by the external arrow of time, our personal spaghetti strand trajectory. They cannot loop back on themselves, but they can divide and merge, meet at points or diverge, sometimes even find themselves cut adrift.

This has important lessons for the technology we use for personal information management: for example we might consider representing threaded time explicitly. Perhaps more critically, all developed nations are facing a crisis of ageing, where dementia and memory loss are key factors for quality of life and cost of care.

The walking man seems far away: a story I was told, more than a memory. Once separated from the day-to-day reminders of walking existence, those external physical representations that we know are critical to us as embodied creatures (Clark 1998; Varela et al. 1991), the thread of the walk becomes separated, floating free, hard to hold on to. How much worse for those whose memory is already threadbare, who are taken into ‘care’ away from the constant reminders of recent life. It is no wonder that the fabric of personality unravels.

## Reweaving

So, our memories lie intertwined, our experiences more like a braid than a single thread; and in physics too we are interwoven, each a strand; we knit together with one another to make this thing we call reality. The three-dimensional cut through four-dimensional space–time that we call ‘now’ is as much an artifice as the map that purports to be the truth on the ground, but is cut across by boundaries and places. And yet for us as social creatures, our lives, our stories interwoven define these places, and the paths that tread place to place create connection from isolation. The threads of our memories are connected through events, the threads of paths connect at places and the journeys we each take, drilling through time and space, meet, when we are fortunate, at moments of human contact, which give points in space significance and make them places, and give our lives significance and make them human.

## References

[CR1] Asimakopoulos S, Dix A (2017). Walking: a grounded theory of social engagement and experience. Interact Comput.

[CR2] Auge M (1995). Non-places: Introduction to an anthropology of supermodernity.

[CR3] Augustine of Hippo (AD 401) The confessions of saint augustine, Book XI. (Tr. E. B. Pusey, 1876). http://www.gutenberg.org/files/3296/3296-h/3296-h.htm

[CR4] Benford S, Giannachi G, Koleva B, Rodden T (2009) From interaction to trajectories: designing coherent journeys through user experiences. In: Proceedings of the SIGCHI conference on human factors in computing systems (CHI’09). ACM, New York, NY, USA, pp 709–718. 10.1145/1518701.1518812

[CR5] Clark A (1998). Being there: putting brain, body and the world together again.

[CR6] Dehaene S, Spelke E, Pinel P, Stanescu R, Tsivkin S (1999). Sources of mathematical thinking: behavioral and brain-imaging evidence. Science.

[CR7] Dix A, Catarci T, Costabile MF, Levialdi S, Santucci G (1996). Closing the Loop: modelling action, perception and information. AVI’96: advanced visual interfaces.

[CR8] Dix A, Pribeanu C, Vanderdonckt J (2002). Managing the ecology of interaction. Proceedings of Tamodia 2002: first international workshop on task models and user interface design (Bucharest, Romania, 18–19 July 2002).

[CR9] Dix A (2009) Paths and patches: patterns of geonosy and gnosis. Chapter 1 in exploration of space, technology, and spatiality: interdisciplinary perspectives. In: Turner P, Turner S, Davenport E (eds) Information science reference, pp 1–16. ISBN: 978-1-60566-020-2. http://alandix.com/academic/papers/paths-and-patches-2009/. Accessed 1 July 2018

[CR10] Dix A (2013a) Alan Walks Wales: one thousand miles of poetry, technology and community. http://alanwalks.wales. Accessed 25 June 2018

[CR11] Dix A (2013b) The Walk: exploring the technical and social margins. Keynote APCHI 2013/India HCI 2013, Bangalore India, 27th September 2013. http://alandix.com/academic/publist-2013.html. Accessed 1 July 2018

[CR12] Dix A, Gill S (2019). Human comprehension of space. Chapter 12 in TouchIT. http://www.physicality.org/TouchIT/. Accessed 1 July 2018

[CR13] Dix A, Wilkinson J, Ramduny D (1998) Redefining organisational memory: artefacts, and the distribution and coordination of work. Workshop on Understanding work and designing artefacts, York, 21st September 1998. http://alandix.com/academic/papers/artefacts98/. Accessed 1 July 2018

[CR14] Dix A, Sheridan J, Lock, S, Ellis G (2004) absenT presence. Position paper for EQUATOR record and reeuse workshop, UCL, London, 12–13 February 2003. http://alandix.com/academic/papers/absent-presence-2004/

[CR15] Harrison S, Dourish P (1996) Re-place-ing space: the roles of place and space in collaborative systems. In: Proceedings of the 1996 ACM conference on computer supported cooperative work, November 16–20, 1996, Boston, Massachusetts, USA, pp 67–76. 10.1145/240080.240193

[CR16] Henrich J, Heine S, Norenzayan A (2010). The weirdest people in the world?. Behav Brain Sci.

[CR17] Hillier B (1996). Space is the machine.

[CR18] Ingold T (2007). Lines: a brief history.

[CR19] Khalid H, Dix A (2010). The experience of photologging: global mechanisms and local interactions. J Pers Ubiquitous Comput.

[CR20] Lakoff G (1987). Women, fire, and dangerous things: what categories reveal about the mind.

[CR21] Lakoff G, Johnsen M (2003). Metaphors we live by.

[CR22] McCarthy J, Wright P (2004). Technology as experience.

[CR23] Parker A (2004). In the blink of an eye: how vision sparked the big bang of evolution.

[CR24] Sobel D (2005). Longitude: the true story of a lone genius who solved the greatest scientific problem of his time.

[CR25] Varela F, Thompson E, Rosch E (1991). The embodied mind: cognitive science and human experience.

